# Variability Predictions for the Next Technology Generations of *n*-type Si_*x*_Ge_1−*x*_ Nanowire MOSFETs

**DOI:** 10.3390/mi9120643

**Published:** 2018-12-05

**Authors:** Jaehyun Lee, Oves Badami, Hamilton Carrillo-Nuñez, Salim Berrada, Cristina Medina-Bailon, Tapas Dutta, Fikru Adamu-Lema, Vihar P. Georgiev, Asen Asenov

**Affiliations:** School of Engineering, University of Glasgow, Glasgow G12 8QW, UK; Jaehyun.Lee@glasgow.ac.uk (J.L.); Oves.Badami@glasgow.ac.uk (O.B.); Hamilton.Carrillo-Nunez@glasgow.ac.uk (H.C.-N.); salim.berrada@glasgow.ac.uk (S.B.); Cristina.MedinaBailon@glasgow.ac.uk (C.M.-B.); Tapas.Dutta@glasgow.ac.uk (T.D.); Fikru.Adamu-Lema@glasgow.ac.uk (F.A.-L.); Asen.Asenov@glasgow.ac.uk (A.A.)

**Keywords:** line edge roughness, metal gate granularity, nanowire, non-equilibrium Green’s function, random discrete dopants, SiGe, variability

## Abstract

Using a state-of-the-art quantum transport simulator based on the effective mass approximation, we have thoroughly studied the impact of variability on ${\mathrm{Si}}_{x}{\mathrm{Ge}}_{1-x}$ channel gate-all-around nanowire metal-oxide-semiconductor field-effect transistors (NWFETs) associated with random discrete dopants, line edge roughness, and metal gate granularity. Performance predictions of NWFETs with different cross-sectional shapes such as square, circle, and ellipse are also investigated. For each NWFETs, the effective masses have carefully been extracted from $sp^{3}d^{5}s^{*}$ tight-binding band structures. In total, we have generated 7200 transistor samples and performed approximately 10,000 quantum transport simulations. Our statistical analysis reveals that metal gate granularity is dominant among the variability sources considered in this work. Assuming the parameters of the variability sources are the same, we have found that there is no significant difference of variability between SiGe and Si channel NWFETs.

## 1. Introduction

Semiconductor fabrication has witnessed amazing progress in the last about 50 to 60 years which has enabled the scaling of the physical dimensions of the metal-oxide-semiconductor field-effect transistors (MOSFETs) at an exponential rate. According to the Institute of Electrical and Electronics Engineers (IEEE) International Roadmap for Devices and Systems (IRDS) report, by the year 2024, the gate length ($L_{G}$) and diameter of transistors are expected to be 10 and 5 nm, respectively, for high-performance logic applications [1]. However, the scaling has slowed down due to increase in a number of detrimental second order effects like source-to-drain tunneling and drain induced barrier lowering (DIBL) [2,3].

In order to overcome these issues, a device with the gate-all-around (GAA) structure is a promising candidate to replace the Fin field-effect transistor (FinFET), which is being adopted in industries [1,4,5]. Devices with the GAA structure showed better electric transport performance thanks to their superior electrostatic integrity. Maheshwaram et al. reported that, by using the vertical GAA Si nanowire MOSFETs (NWFETs) instead of the FinFET, the ring oscillator delay and the power consumption are improved by 33% and 45%, respectively [6]. In addition, nanowires based on different materials and geometry cross-section can be used as transducers, sensors or photovoltaic devices [7,8,9,10].

Studying on the channel material engineering as well as the gate structure is very important to overcome the short channel effects in nanoscale devices. SiGe, III-V, and two-dimensional materials such as graphene and transition metal dichalcogenide are attracting attention as the channel material in future devices thanks to their small transport effective masses ($m_{trans}^{*}$) [3,11,12,13,14]. It is noteworthy that materials with smaller $m_{trans}^{*}$ can contribute to increases ON-state current ($I_{\mathrm{ON}}$) but increases OFF-sate current ($I_{\mathrm{OFF}}$) as well in the short channel device due to the source-to-drain tunneling currents. Moreover, transistors with small band-gap materials are suffering from the band-to-band leakage currents [13]. Unfortunately, the overwhelmingly superior material that can replace Si has not been found yet. In this paper, we concentrate on SiGe, which is more compatible with the current complementary metal-oxide-semiconductor (CMOS) technology [15]. In addition, material properties of SiGe can be adjusted by the mole fraction to have the advantages of Si and Ge together.

Previous simulation studies have shown that random discrete dopants (RDD), line edge roughness (LER), and metal gate granularity (MGG) induce significant variability in ultra-scaled InGaAs [16] and Si [17,18] channel nanoscale devices. However, the former used classical transport models, whereas the latter considered a very small number of statistical samples due to the computational cost of quantum transport simulations. To the best of our knowledge, a study comparing the impact of different sources of variability of SiGe channel NWFETs using the quantum transport simulations with a large number of samples is missing.

In this paper, we focus on the investigation of the impact of dominant sources of statistical variability (RDD, LER and MGG) in *n*-type ${\mathrm{Si}}_{x}{\mathrm{Ge}}_{1-x}$ channel GAA NWFETs with different cross-section shapes. In order to capture the source-to-drain tunneling in the nanoscale devices, the quantum transport problem for electrons is solved within the parabolic effective mass (PEM) approximation by means of the non-equilibrium Green’s function (NEGF) formalism implemented in the Glasgow Nano-Electronic Simulation Software (NESS) [19]. We also confirm that the calibrated confinement and transport effective masses can reproduce the empirical tight binding (ETB) band structures. For a reliable statistical analysis, an ensemble of 200 transistor samples for each set of variability sources has been adopted. All together, we have performed approximately 10,000 quantum transport simulations with 7200 different transistor samples.

The paper is organized as follows. In Section 2, we discuss the details related to the generation of the statistical variability sources such as RDD, MGG and LER, implementation of the NEGF formalism and the effective mass extraction method from $sp^{3}d^{5}s^{*}$ ETB band structure calculations. This is followed by the discussion of the simulation results in Section 3. Finally, we summarize our results in Section 4.

## 2. Simulation Framework

### 2.1. Device Structure with the Variability Sources Included

**Figure 1:**
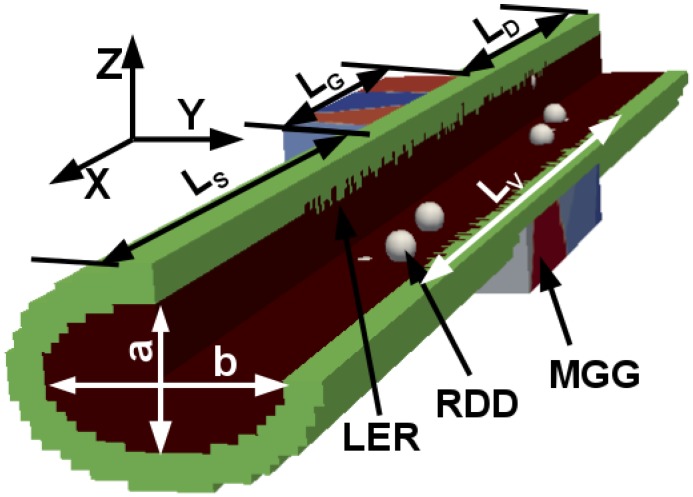
Schematic diagram of the elliptical gate-all-around nanowire metal-oxide-semiconductor field-effect transistors (GAA NWFET) (a = 3 nm and b = 5 nm) highlighting variability sources. For the square and circular nanowires (NWs), a = b = 5 nm. $L_{S}$ = $L_{D}$ = 28 nm, $L_{G}$ = 10 nm, and $L_{V}$ = 26 nm. The doping concentrations in source/drain and channel regions are 10^20^ (*n*-type) and 10^15^ (*p*-type) cm^−3^, respectively. RDD–random discrete dopants, LER–line edge roughness and MGG–metal gate granularity.

Figure 1 illustrates the schematic diagram of GAA NWFETs with an elliptic cross-sectional shape. The three dominant variability sources including RDD, LER, and MGG are also highlighted in Figure 1. RDD and LER are generated in the channel region (10.0 nm) and in equal portions of the source and the drain (8.0 nm each), resulting in $L_{v}$ = 26.0 nm. The remaining source and drain regions (20.0 nm each) are assumed to have continuous doping profile in order to ensure good convergence of the electrostatic potential.

For the generation of RDD, a rejection technique has been adopted by considering the atomic arrangement in ${\mathrm{Si}}_{x}{\mathrm{Ge}}_{1-x}$ NW crystal structures [20] with the corresponding lattice parameter. We generate a random number between 0 and 1 in each atom of ${\mathrm{Si}}_{x}{\mathrm{Ge}}_{1-x}$, and substitute the atom with a dopant atom if the random number is less than the criteria (CR). The criteria can be written as:

(1) $$CR=N_{D}V_{atom},$$

where $N_{D}$ is the doping density at this site and $V_{atom}$ is the volume of the corresponding atom. Because $V_{atom}$ is a constant value determined by the lattice parameter, as $N_{D}$ increases, the probability that a dopant atom is located increases. Therefore, the total number of dopant atoms follows the Poisson distribution [21].

LER at the interface between ${\mathrm{Si}}_{x}{\mathrm{Ge}}_{1-x}$ and gate oxide is characterized by an auto-correlation function [22]:

(2) $$C\left(r\right)=\Delta_{m}^{2}e^{-\sqrt{2}r/L_{m}},$$

where $\Delta_{m}$ is the root mean square, $L_{m}$ is the correlation length, and *r* is the length between two points. Herein, $\Delta_{m}$ and $L_{m}$ are 0.2 and 1.0 nm, respectively, which is consistent with experimental data for Si [23]. To be consistent, we have used the same value of these parameters for ${\mathrm{Si}}_{x}{\mathrm{Ge}}_{1-x}$ channel devices.

Regarding MGG, the grains in the TiN metal gate region are generated by using the Voronoi algorithm [24,25]. The value of the work-function for each grain can be either 4.4 or 4.6 eV with the probability of 40% or 60% based on previous experimental results [26]. It was reported that, as the grain size increases, the more significant variability is observed, meaning that the small average grain size causes less variability [24]. Therefore, the average grain size of 3.0 nm used in this paper is small enough to expect a relatively less MGG-induced variability.

Following the IRDS specifications for the node “4/3” [1], the *n*-type ${\mathrm{Si}}_{x}{\mathrm{Ge}}_{1-x}$ (*x* = 1.0, 0.8, 0.5, and 0.2) channel GAA NWFETs with $L_{G}$ = 10.0 nm and a diameter (or width) of 5.0 nm (see Figure 1) are considered. NWFETs with square, circle, and elliptic cross-sectional shapes are also studied and their corresponding cross-section dimensions are chosen to have the same footprint to keep the technology node. Indeed, NWFETs with elliptic cross-sectional shapes can be referred to the nano-sheet MOSFETs [27]. The transport direction in all the devices is along [100]. The equivalent oxide thickness is 0.8 nm. The source-to-drain bias $V_{\mathrm{DS}}$ is set to 0.6 V. All simulations are performed at 300 K.

### 2.2. Quantum Transport Formalism

The electron quantum transport problem is solved by exploiting the coupled mode NEGF formalism with the PEM Hamiltonian [28]. Assuming steady-state conditions, we briefly summarize the main features of the NEGF approach in matrix notation. Within the PEM approximation, the discretized mode-space Green’s function is defined as

(3) $$G_{\nu }^{r}\left(E\right)={\left[EI-H_{\nu }-\Sigma_{L,\nu }^{r}\left(E\right)-\Sigma_{R,\nu }^{r}\left(E\right)\right]}^{-1},$$

where *I* is the identity matrix and $H_{\nu }$ represents the mode-space version of the Hamiltonian for the ν th conduction band valley. $\Sigma_{L/R}^{r}$ is the retarded self-energy for the left/right semi-infinite device contact, usually being computed by adopting the recursive algorithm proposed in Ref [29].

The lesser and greater Green’s functions are then obtained from

(4) $$G_{\nu }^{≶}=G_{\nu }^{r}\left(\Sigma_{L,\nu }^{≶}+\Sigma_{R,\nu }^{≶}\right)G_{\nu }^{r†}$$

with lesser ($\Sigma^{<}$) and greater self-energies ($\Sigma^{>}$). They are related to their corresponding retarded counterpart by

(5) $$\Sigma^{r}=\frac{1}{2}\left(\Sigma^{>}-\Sigma^{<}\right),$$

where the energy variable *E* has been omitted for brevity. In practice, the real part of the retarded self-energy in Equation (5) is neglected. This approximation shall not introduce significant error in the transport properties [30]. Once the lesser and greater Green’s functions are known, physical quantities such as carrier density and current can be computed respectively as,

(6) $$\begin{array}{cc} n(x_{j},y,z) & =-i\times 2\sum_{\nu }\sum_{n,m}\int \frac{dE}{2\pi }G_{nm}^{<}\left(x_{j},x_{j};E\right)\phi_{n}\left(y,z;x_{j}\right)\phi_{m}^{†}\left(y,z;x_{j}\right), \end{array}$$

(7) I(xj)=−2×eℏ∑ν∑n,m∫dE2π2ReHnm,ν(xj,xj+1)Gmn<(xj+1,xj;E),

in which the factor 2 considers the spin degeneracy. The eigenfunction $\phi_{n}\left(y,z;x_{j}\right)$ for the mode *n* is calculated by solving the 2D Schrödinger equation corresponding to the cross-section plane at $x_{j}$. In nanostructures, such as the nanowires considered in this paper, only few low energy modes are necessary due to the strong confinement. Therefore, there is a significant gain in the size of the matrices that must be inverted in the recursive algorithm [30] employed in NESS for computing the diagonal and off-diagonal elements of $G^{<}$ in Equations (6) and (7), respectively. Finally, Equation (6) is self-consistently coupled to Poisson equation. When the convergence criterion for the electrostatic potential is reached, the current is then calculated from Equation (7).

### 2.3. Extraction of Effective Masses

**Figure 2:**
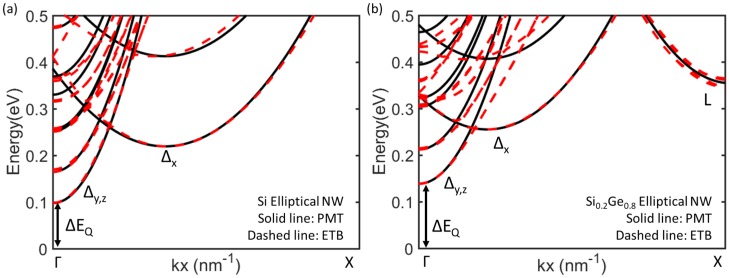
Band structures of (**a**) Si and (**b**) ${\mathrm{Si}}_{0.2}{\mathrm{Ge}}_{0.8}\;5\times 3$ nm^2^ elliptical NWs. The bulk conduction band edge is set to 0.0 eV. $\Delta E_{Q}$ is also remarked.

In order to model the conduction band for the transport simulation, the PEM Hamiltonian is adopted with transport and confinement effective masses extracted from $sp^{3}d^{5}s^{*}$ ETB method with Boykin’s parameter set, implemented in Synopsys QuantumATK [31,32]. For ${\mathrm{Si}}_{x}{\mathrm{Ge}}_{1-x}$ materials, virtual crystal approximation is used [33]. Figure 2 shows the conduction band structures of Si and ${\mathrm{Si}}_{0.2}{\mathrm{Ge}}_{0.8}$ NWs as an example. It is highlighted that *L*-valley is observed in ${\mathrm{Si}}_{0.2}{\mathrm{Ge}}_{0.8}$ NW but not in Si NW. Moreover, it is found that the quantization energy ($\Delta E_{Q}$), the energy difference of conduction band edges of bulk and NW, of ${\mathrm{Si}}_{0.2}{\mathrm{Ge}}_{0.8}$ NW is larger than that of Si NW.

The transport effective masses are directly calculated from the ETB band structures as follows:

(8) $$m_{trans}^{*}=\hbar^{2}{\left(\frac{\partial^{2}E}{\partial k_{x}^{2}}\right)}^{-1}.$$

The extraction of confinement effective masses ($m_{conf}^{*}$) is more complicated. The least-squares method is used to find the best value of $m_{conf}^{*}$ to fit $\Delta E_{Q}$ and the energy gap between the first and the second conduction sub-band energies ($\Delta E_{sub}$) as follows:

(9) $$S={\left(\Delta E_{Q}^{ETB}-\Delta E_{Q}^{PEM}\right)}^{2}+{\left(\Delta E_{sub}^{ETB}-\Delta E_{sub}^{PEM}\right)}^{2},$$

where $\Delta E_{Q}^{ETB}$ ($\Delta E_{sub}^{ETB}$) and $\Delta E_{Q}^{PEM}$ ($\Delta E_{sub}^{ETB}$) are $\Delta E_{Q}$ ($\Delta E_{sub}$) obtained from *ETB* and *PEM* methods, respectively. It is noteworthy that $\Delta E_{Q}^{PEM}$ and $\Delta E_{sub}^{ETB}$ are the function of $m_{conf}^{*}$. Herein, minimized the squared residue *S* indicates $m_{conf}^{*}$ are well extracted. As a result, the *PEM* method successfully reproduces the *ETB* conduction band structures. The extracted $m_{trans}^{*}$ and $m_{conf}^{*}$ are summurized in Table 1.

## 3. Simulation Results and Discussion

**Figure 3:**
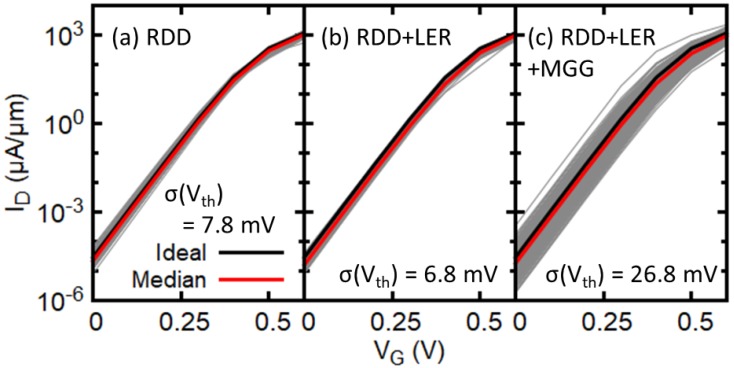
Transfer characteristics of ${\mathrm{Si}}_{0.2}{\mathrm{Ge}}_{0.8}$ elliptical GAA NWFETs associated with (**a**) random discrete dopants (RDD), (**b**) RDD and line edge roughness (LER) and (**c**) RDD, LER and metal gate granularity (MGG. The ideal device refers to a device with continuous and uniform doping profiles in the source and drain and no variability sources. Corresponding standard deviation of $V_{\mathrm{th}}\;\sigma (V_{\mathrm{th}})$ is also indicated. $V_{\mathrm{DS}}$ = 0.6 V.

Figure 3 shows the statistical transfer characteristics for ${\mathrm{Si}}_{0.2}{\mathrm{Ge}}_{0.8}$ channel elliptical GAA NWFETs considering different sets of statistical variability sources. The drain current is normalized by the diameter (width) of 5 nm of NWFETs. A statistical ensemble of 200 devices has been used in this work. Significant statistical variability is observed in terms of $I_{\mathrm{ON}}$, $I_{\mathrm{OFF}}$ and threshold voltage ($V_{\mathrm{th}}$). Figure 3a,b show that the change in RDD-induced variability when adding LER is small, whereas Figure 3c clearly shows that MGG is the dominant source of variability in the devices under consideration although very small average grain size of 3.0 nm is used. It is also found that the median of subthreshold slope (SS) with RDD, LER, and MGG is 62.8 mV/dec, which is comparable to the value of SS (63.0 mV/dec) for the corresponding ideal device. Standard deviation of SS is 0.78 mV/dec suggesting that SS does not change much due to the impact of statistical variability sources.

**Figure 4:**
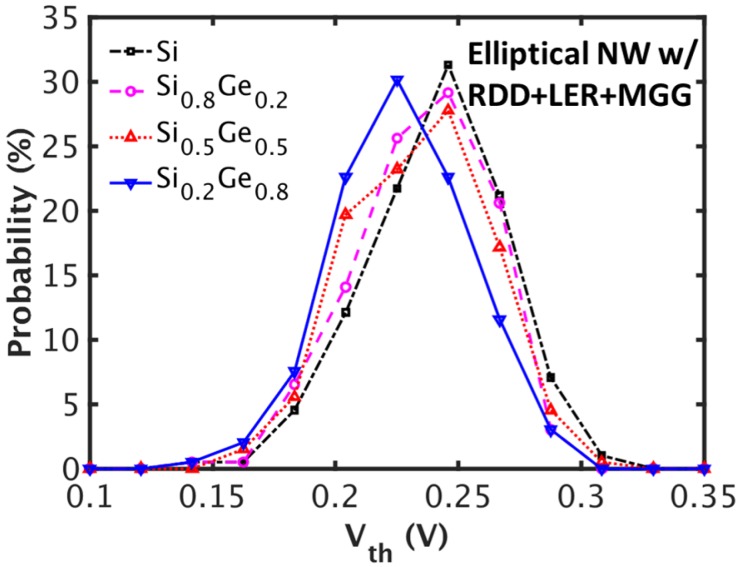
Distributions of threshold voltage ($V_{\mathrm{th}}$) for the elliptical NWFETs with different mole fractions. RDD, LER, and MGG are taken into account.

Figure 4 shows the probability distribution of $V_{\mathrm{th}}$ with RDD, LER, and MGG. There is a shift in the median, but the distribution shapes (bell shapes) and standard deviations are similar regardless of the mole fraction. Similar qualitative results for the combination of other architectures and materials are observed.

Medians of $I_{\mathrm{ON}}$ and $I_{\mathrm{OFF}}$ of all simulated devices considered are summarized in Table 2. Herein, $I_{\mathrm{ON}}$ is defined at $V_{\mathrm{DS}}$ = $V_{\mathrm{GS}}$ = 0.6 V and $I_{\mathrm{OFF}}$ is defined at $V_{\mathrm{DS}}$ = 0.6 V and $V_{\mathrm{GS}}$ = 0.0 V. Variation in $I_{\mathrm{OFF}}$ is significant with respect to the Ge mole fraction as compared to $I_{\mathrm{ON}}$, but all $I_{\mathrm{OFF}}$ satisfy the IRDS criterion of staying below 100 nA/μm [1].

**Figure 5:**
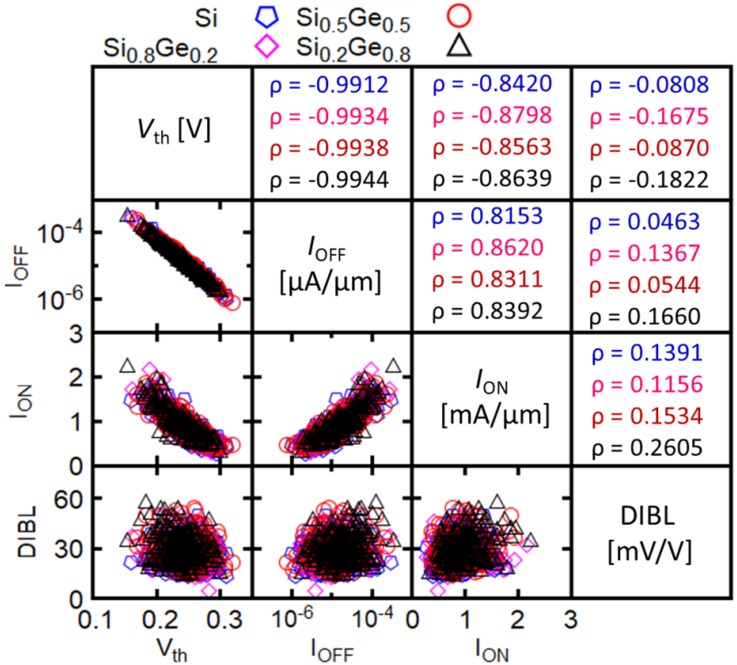
Correlation between important FoMs for the elliptical GAA NWFETs with different Ge mole fraction. The bottom left of the table shows correlation scatter plots and the top right shows correlation coefficients which are also listed in the following order: Si (blue), ${\mathrm{Si}}_{0.8}{\mathrm{Ge}}_{0.2}$ (magenta), ${\mathrm{Si}}_{0.5}{\mathrm{Ge}}_{0.5}$ (red), and ${\mathrm{Si}}_{0.2}{\mathrm{Ge}}_{0.8}$ (black).

Figure 5 summarizes the correlations between important figures-of-merits (FoMs) in terms of scatter plots and correlation coefficients: $I_{\mathrm{ON}}$, $I_{\mathrm{OFF}}$, $V_{\mathrm{th}}$ and DIBL. Herein, $V_{\mathrm{th}}$ is calculated using the constant current method with the current criteria $I_{\mathrm{th}}$ = 100 nA/μm. As data in Figure 5 shows, the correlation coefficients ρ for the different Ge mole fraction are comparable and very similar to those for Si. In addition, as expected, the $V_{\mathrm{th}}$ and $I_{\mathrm{OFF}}$ show negative correlation with ρ almost equal to 1. Negatively correlated are $I_{\mathrm{ON}}$ and $V_{\mathrm{th}}$ with ρ which still has very high value (around −0.85) but less than the ρ value between the $V_{\mathrm{th}}$ and $I_{\mathrm{OFF}}$. $I_{\mathrm{OFF}}$ and $I_{\mathrm{ON}}$ show positive correlation with a correlation coefficient close to 0.85. As expected, the DIBL parameter is not correlated to any of the other FoMs, as shown by the value of ρ very close to 0. Hence, our results suggest that replacing Si channel by ${\mathrm{Si}}_{x}{\mathrm{Ge}}_{1-x}$ channel will not solve the variability issues in sub-10 nm gate-length NWFETs.

**Figure 6:**
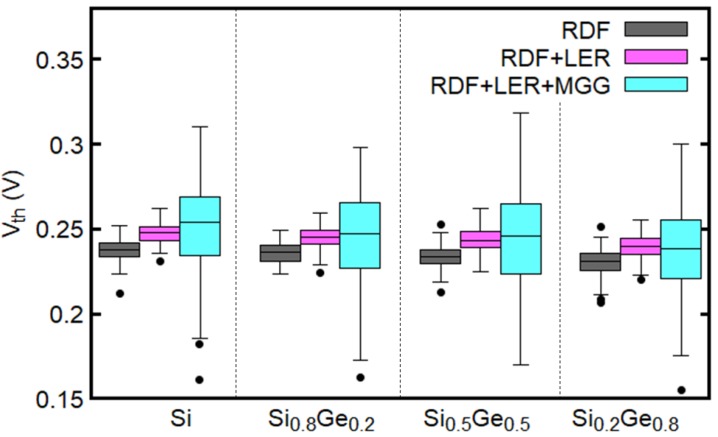
Dependence of $V_{\mathrm{th}}$ of the elliptical GAA NWFETs on the variability sources and the Ge mole fraction.

Figure 6 shows the variation of $V_{\mathrm{th}}$ for elliptical GAA NWFETs with different Ge mole fractions and different sets of variability sources. It is found that, despite the small average grain size of 3 nm, MGG is the dominant source of variability in the considered devices regardless of the Ge mole fraction. Moreover, the median of $V_{\mathrm{th}}$ increases as the number of the variability sources included in the simulations increases. We have also found that, as the Ge mole fraction increases, $V_{\mathrm{th}}$ decreases. This can be attributed to the increase in the contribution of the *L*-valley (see Figure 2) [34]. Therefore, the ${\mathrm{Si}}_{0.2}{\mathrm{Ge}}_{0.8}$ channel devices have larger $I_{\mathrm{ON}}$ than the Si devices considered in this paper as shown in Table 2.

**Figure 7:**
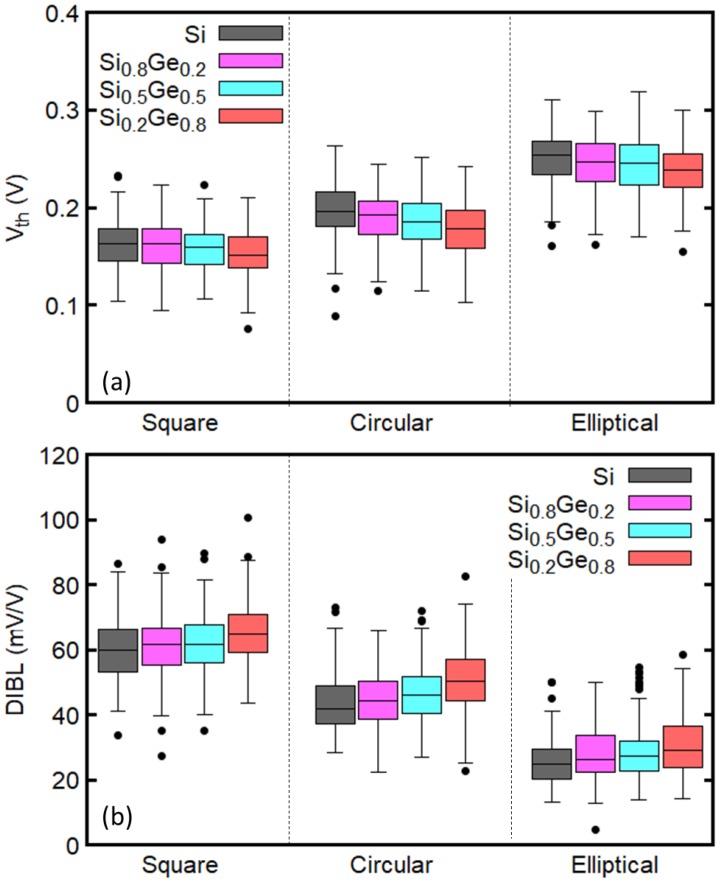
Dependence of (**a**) $V_{\mathrm{th}}$ and (**b**) drain induced barrier lowering (DIBL) on the Ge mole fraction and cross-sectional shape. RDD, LER, and MGG are considered

Figure 7a shows the variation of $V_{\mathrm{th}}$ for GAA NWFETs with different mole fractions of Ge and different cross-sectional shapes considering the effects of RDD, LER, and MGG. The Ge mole fraction and the shape of the cross-section do not have significant effect on $V_{\mathrm{th}}$ variability. Regardless of the cross-sectional shapes, $V_{\mathrm{th}}$ is smaller for the larger mole fraction of Ge, which is in a good agreement with the results in Figure 6. Additionally, it is found that the median of $V_{\mathrm{th}}$ decreases when the cross-sectional shape is changed from ellipse to circle and to square, in this order. This trend is consistent with the dependence of $V_{\mathrm{th}}$ on the inverse of the cross-sectional area, which increases in the aforementioned order. Therefore, the elliptical devices have smaller $I_{\mathrm{ON}}$ than the other devices (see Table 2).

The variation of DIBL is plotted in Figure 7b. DIBL calculated from the ideal device is underestimated with respect to its median when considering variability sources (see Table 3). It is interesting to note that ${\mathrm{Si}}_{0.2}{\mathrm{Ge}}_{0.8}$ channel devices with larger $I_{\mathrm{ON}}$ (see Table 2) also show larger DIBL than others, regardless of the cross-sectional shape. Furthermore, the median and the variation of DIBL of the elliptical devices are smaller than that of square and circular devices.

## 4. Conclusions

We have performed a comprehensive variability analysis of *n*-type ${\mathrm{Si}}_{x}{\mathrm{Ge}}_{1-x}$ (x = 1.0, 0.8, 0.5, and 0.2) channel GAA NWFETs using 7200 samples. The electron transport has been modeled by means of the coupled-mode space NEGF formalism implemented in NESS. Our results show that the Ge mole fraction and cross-sectional shapes do not affect significantly the variability in GAA NWFETs, and MGG is the dominant source of variability as when compared to RDD and LER. It is noticeable that the small average grain size of 3 nm is considered in this paper, which is expected to cause relatively less MGG-induced variability. We have also found that ${\mathrm{Si}}_{0.2}{\mathrm{Ge}}_{0.8}$ channel devices have not only smaller $V_{\mathrm{th}}$ but also larger DIBL compared to the devices with lower Ge mole fractions indicating that they suffer the most from short channel effects. In addition, elliptical GAA NWFETs have smaller DIBL compared to square and circular devices, while providing smaller $I_{\mathrm{ON}}$.

## Figures and Tables

**Table 1:** Calculated effective masses of Si and ${\mathrm{Si}}_{x}{\mathrm{Ge}}_{1-x}$ nanowires (NWs) with various cross-sectional shapes. Herein, unit is $m_{0}$, the rest electron mass.

		Degeneracy	Square			Circle			Ellipse		
			$m_{x}$	$m_{y}$	$m_{z}$	$m_{x}$	$m_{y}$	$m_{z}$	$m_{x}$	$m_{y}$	$m_{z}$
Si	$\Delta_{x}$	2	0.918	0.240	0.240	0.915	0.224	0.224	0.927	0.464	0.146
	$\Delta_{y}$	2	0.233	0.953	0.237	0.236	0.887	0.215	0.241	0.839	0.220
	$\Delta_{z}$	2	0.233	0.242	0.875	0.236	0.208	0.896	0.241	0.206	0.886
${\mathrm{Si}}_{0.8}{\mathrm{Ge}}_{0.2}$	$\Delta_{x}$	2	0.861	0.235	0.235	0.849	0.287	0.287	0.875	0.321	0.198
	$\Delta_{y}$	2	0.240	0.884	0.221	0.235	1.342	0.262	0.251	0.757	0.224
	$\Delta_{z}$	2	0.240	0.220	0.885	0.235	0.259	1.366	0.251	0.192	0.905
${\mathrm{Si}}_{0.5}{\mathrm{Ge}}_{0.5}$	$\Delta_{x}$	2	0.799	0.241	0.241	0.788	0.286	0.286	0.818	0.392	0.179
	$\Delta_{y}$	2	0.250	0.864	0.224	0.247	1.042	0.272	0.268	0.674	0.210
	$\Delta_{z}$	2	0.250	0.224	0.816	0.247	0.270	1.015	0.268	0.194	0.809
${\mathrm{Si}}_{0.2}{\mathrm{Ge}}_{0.8}$	$\Delta_{x}$	2	0.759	0.237	0.237	0.739	0.285	0.285	0.788	0.448	0.174
	$\Delta_{y}$	2	0.266	0.788	0.217	0.258	0.952	0.272	0.286	0.657	0.206
	$\Delta_{z}$	2	0.266	0.213	0.798	0.258	0.272	0.958	0.286	0.186	0.828
	*L*	4	0.350	0.134	0.297	0.500	0.147	0.449	0.600	0.327	0.152

**Table 2:** Medians of $I_{\mathrm{ON}}$ and $I_{\mathrm{OFF}}$ for the ${\mathrm{Si}}_{x}{\mathrm{Ge}}_{1-x}$ nanowire metal-oxide-semiconductor field-effect transistors (NWFETs). Random discrete dopants (RDD), line edge roughness (LER), and metal gate granularity (MGG) are considered.

${\mathrm{Si}}_{x}{\mathrm{Ge}}_{1-x}$	$I_{\mathrm{ON}}$ (mA/μm)/$I_{\mathrm{OFF}}$ (pA/μm)		
	Square	Circular	Elliptical
Si	1.59/397	1.37/98.9	0.771/9.26
${\mathrm{Si}}_{0.8}{\mathrm{Ge}}_{0.2}$	1.71/427	1.50/127	0.862/11.7
${\mathrm{Si}}_{0.5}{\mathrm{Ge}}_{0.5}$	1.70/473	1.51/151	0.861/12.7
${\mathrm{Si}}_{0.2}{\mathrm{Ge}}_{0.8}$	1.84/668	1.63/210	0.958/18.1

**Table 3:** The comparison of drain induced barrier lowering (DIBL) in ${\mathrm{Si}}_{0.2}{\mathrm{Ge}}_{0.8}$ channel devices obtained from the ideal devices and statistical simulations.

Cross-Sectional Shape (RDD + LER + MGG)	Ideal Device	Median
Square	62.4 mV/V	64.7 mV/V
Circle	42.8 mV/V	50.2 mV/V
Ellipse	20.3 mV/V	29.2 mV/V
